# A rare case of synchronous colocolic intussusception in association with Peutz–Jeghers syndrome

**DOI:** 10.1259/bjrcr.20150314

**Published:** 2016-07-25

**Authors:** Om Biju Panta, Santosh Maharjan, Sujan Manandhar, Sharma Paudel, Ram Kumar Ghimire

**Affiliations:** ^1^Department of Radiology and Imaging, Tribhuwan University Teaching Hospital, Kathmandu, Nepal; ^2^Gastrointestinal Surgery Unit, Department of Surgery, Tribhuwan University Teaching Hospital, Kathmandu, Nepal

## Abstract

Adult intussusception is rare and is almost always associated with a lead point. Polyposis syndromes are a common cause of adult intussusceptions, with polyps acting as lead points. Peutz–Jeghers syndrome is associated with benign hamartomatous polyps and mucocutaneous pigmentation. Although hamartomatous polyps are not premalignant, there is an increased risk of gastrointestinal and non-gastrointestinal malignancy, most commonly involving the small bowel. Most patients with Peutz–Jeghers syndrome with acute abdomen are diagnosed to have intussusceptions, mostly of the enteroenteric type. Colocolic intussusceptions are rare in Peutz–Jeghers syndrome. To the best of our knowledge, synchronous colocolic intussusception in association with Peutz–Jeghers syndrome has not been previously reported. Here we present a case of malignant jejunal mass and synchronous colocolic intussusceptions in a patient with Peutz–Jeghers syndrome.

## Summary

Peutz–Jeghers syndrome is an autosomal dominant disorder characterized by multiple gastrointestinal hamartomatous polyps associated with hyperpigmentation of the lips and oral mucosa. Hamartomatous polyps are not considered premalignant; however, there is an increased risk of gastrointestinal and non-gastrointestinal malignancy in Peutz–Jeghers syndrome. Malignancy most commonly occurs within the small bowel.^1^

Peutz–Jeghers polyp may act as a lead point for intussusceptions; however, the complication is almost exclusively limited to the small bowel.^1^ Colocolic intussusceptions in association with Peutz–Jeghers syndrome is a rare occurrence. To the best of our knowledge, synchronous colocolic intussusception in patients with Peutz–Jeghers syndrome has not been reported before. Here we present a case of Peutz–Jeghers syndrome with a malignant jejunal mass and two synchronous colocolic intussusceptions in a 17-year-old male.

## Case report

**Figure 1.**
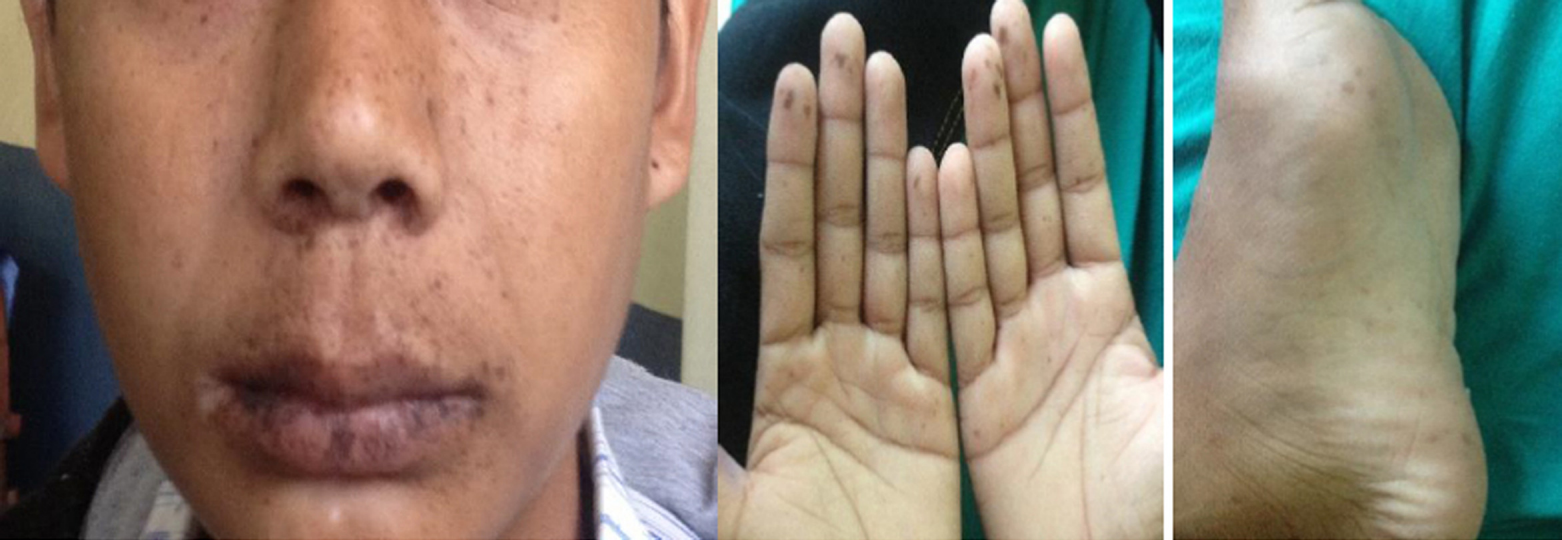
Images showing hyperpigmented macules on the lips, palms and sole.

A 17-year-male patient presented to the emergency department of Tribhuvan University Teaching Hospital with acute onset of abdominal pain of 1-week duration. There was a past history of laparotomy for an acute abdomen; however, no documentation was available. His mother and sister also had pigmented macules on the lips; however, no family history of abdominal complaints was present. On examination, there were multiple hyperpigmented macules on the lips, palms and soles (Figure 1). Systemic examinations, including abdominal examination, were unremarkable apart from diffuse abdominal tenderness on palpation. Ultrasound examination performed at the emergency department showed a large mass in the periumbilical region.

**Figure 2.**
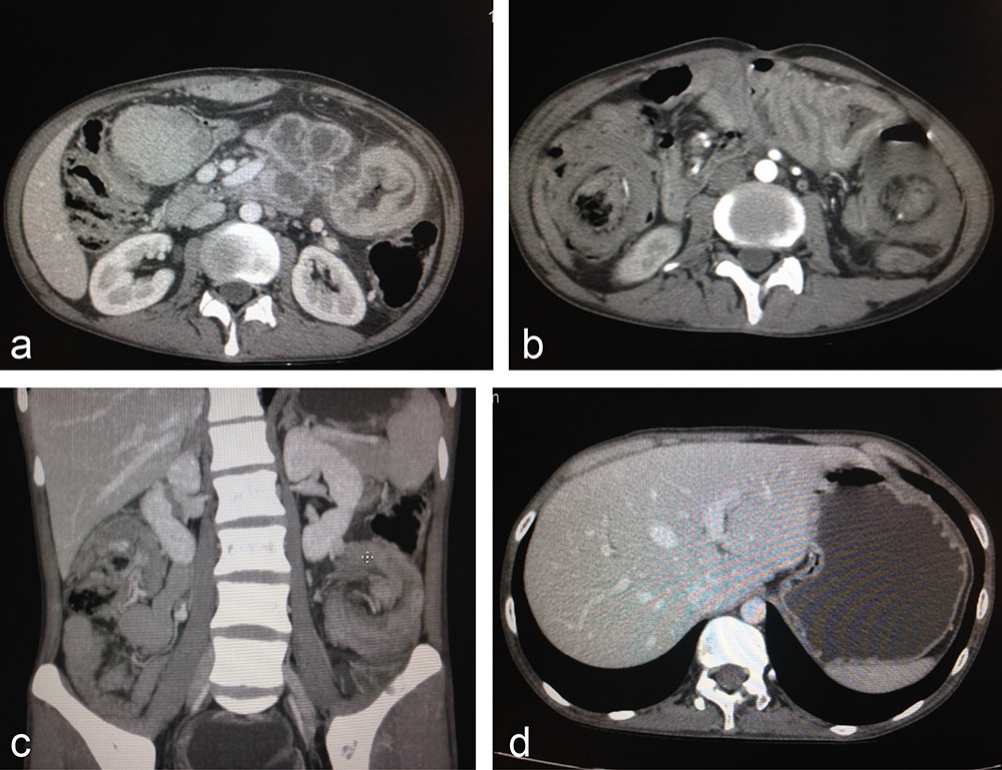
(a) Axial CT scan showing a heterogeneous peripherally enhancing mass in the mesentery just above the level of the renal hilum; the mass was not separate from the jejunal loops and was abutting the superior mesenteric artery, with maintained fat plane. (b) Reformatted oblique axial CT scan showing telescoping of the descending colon into the sigmoid colon, and the caecum into the ascending colon; however, the invaginated loop showed normal enhancement. (c) Coronal reformatted CT scan showing telescoping of the descending colon into the sigmoid colon, and the caecum into the ascending colon; however, the invaginated loop showed normal enhancement. (d) Axial CT image demonstrating multiple enhancing polyps in the stomach.

A CT scan was performed to further characterize the mass. The CT scan showed a heterogeneous peripheral enhancing mass in the mesentery just above the level of the renal hilum, which was not separate from the jejunal loops (Figure 2). The mass was abutting the superior mesenteric artery, with maintained fat plane. The mass was considered malignant on the CT scan because of heterogeneous enhancement, and infiltration of the mesentery and the jejunal loops. Telescoping of the descending colon into the sigmoid colon, and the caecum into the ascending colon was also noted; however, the invaginated loop showed normal enhancement. A large, enhancing polypoid mass was noted at the tip of the invaginated bowel loops at the two sites acting as lead points (Figure 2b,c). The finding suggested an imaging diagnosis of synchronous colocolic intussusception. Multiple intraluminal polyps were also noted in the stomach, jejunum and colon (Figure 2d). Endoscopic evaluation performed at a later date also showed multiple polyps in the stomach and duodenum.

A diagnosis of Peutz–Jeghers syndrome with a malignant mass in the jejunum, multiple intestinal polypoid masses and two synchronous colocolic intussusceptions was made based on imaging and clinical findings. The differential diagnoses included synchronous colonic malignancy with synchronous colocolic intussusception and familial adenomatous polyposis with synchronous colonic malignancy. However, the presence of multiple pigmented macules and polyps in the stomach and bowel loops were more in favour of a polyposis syndrome and Peutz–Jeghers syndrome in particular. A family history of similar pigmented macules consolidated the diagnosis further.

**Figure 3.**
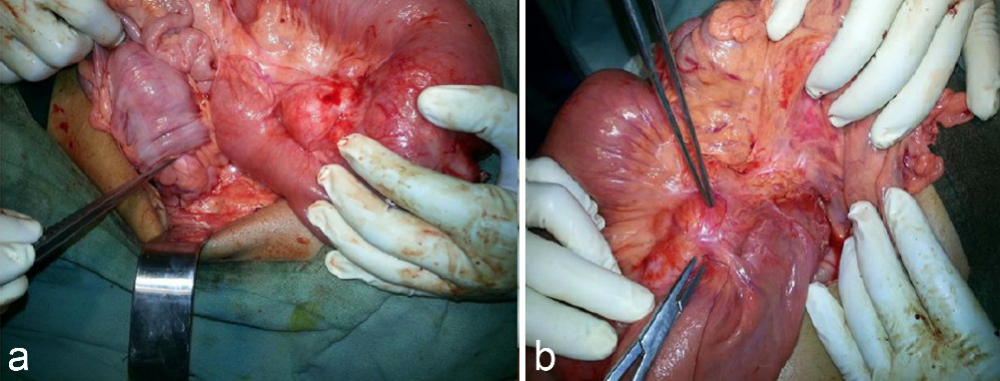
(a) Intraoperative photograph demonstrating the colocolic intussusceptions, which were reduced. (b) Intraoperative photograph demonstrating a nodular mass in the jejunum infiltrating into the root of the superior mesenteric artery.

The patient was posted for laparotomy, which confirmed the CT scan findings and demonstrated a mass in the jejunum, which was infiltrating into the root of the superior mesenteric artery; the mass was unresectable and thus biopsied. Also synchronous colocolic intussusceptions were confirmed, which were reduced (Figure 3a,b). Scar was noted in the jejunal loops, suggesting previously resected bowel loops. The surgical findings confirmed the CT scan diagnosis. Histopathology of the mass found in the jejunum and mesentery showed nests, sheets and ill-formed glands of markedly pleomorphic tumour cells. The tumour cells had abundant amount of clear to vacuolated eosinophilic cytoplasm with enlarged nucleus and prominent nucleoli. Apart from that, multiple multinucleated and bizarre cells were also seen. Some amount of necrosis was noted in the centre. Four mitotic figures were seen per high power field. These findings favoured a poorly differentiated malignant tumour and a differential diagnosis of poorly differentiated jejunal adenocarcinoma was made. The patient was referred to the oncology unit to begin chemotherapy.

## Discussion

Adult bowel intussusception is a rare but challenging condition for the surgeon. Pre-operative diagnosis is usually missed or delayed because of non-specific and often subacute symptoms, without the pathognomonic clinical picture.^2^ Contrary to the paediatric age group, diagnosing intussusceptions in adults preoperatively based on clinical features alone is difficult and imaging plays a crucial role. Most studies also indicate that CT scan is the most accurate pre-operative diagnostic tool for intussusceptions with a diagnostic accuracy of approximately 83%.^3^ CT scan may also define the location, nature of the mass and its relationship to the surrounding tissues; additionally, it may help in disease staging in patients in whom malignancy is the suspected cause of intussusceptions.^4^

Hamartomatous polyp in Peutz–Jeghers syndrome can act as a lead point for intussusception, which commonly involves the small bowel and occurs during adolescence.^1^ Although rare, multiple synchronous intussusceptions have also been reported in the literature in patients with Peutz–Jeghers syndrome, with the enteroenteric type being the most common.^5^

Synchronous colocolic intussusception is extremely rare and has been reported by Chen et al^4^ in 2012 in association with synchronous adenocarcinoma in the proximal transverse and sigmoid colon. To the best of our knowledge, no other case report of synchronous colocolic intussusceptions has been reported in the literature and none in association with Peutz–Jeghers syndrome. The presence of intussusception in adults should always ring the bell for the presence of a lead point. With synchronous intussusception, there must be multiple lead points, making polyposis syndrome the leading cause of synchronous intussusception. However, synchronous intussusception is very rare even in polyposis syndromes, rather recurrent intussusception tends to occur, making screening a very important modality in these syndromes.^4,5^ Also, colonic intussusception is associated more frequently with a malignant tumour, while small bowel intussusception is associated more frequently with benign lesions.^6^

Hamartomatous polyps are considered benign; however, rare cases of malignancy developing in these polyps has been reported.^7,8^ The polyps can develop in any part of the gastrointestinal tract; however, the prevalence of these polyps in order of frequency is in the jejunum, ileum, duodenum, colon and stomach. The polyps may vary from 1 mm to 5 cm in size and are usually pedunculated with a coarse lobulated surface. Rarely, polyps may also be seen in the nostrils, lungs, renal pelvis and urinary bladder.^1^

Mucocutaneous hyperpigmentation is seen on the lips, around the mouth, eyes, nostrils, on the buccal surface, and rarely on the palms and soles. These hyperpigmented macules contain melanotic deposits and are considered hamartomatous in origin without the potential for malignant transformation. These macules are prominent during infancy and childhood, and may fade away during puberty and adulthood.^9^

The syndrome usually presents with complications in the first two decades of life, with the median age of presentation being 11–13 years.^10^ Half of the patients will have experienced symptoms by the age of 20 years. Patients most commonly present with an acute abdomen owing to intussusceptions, with 50% of patients experiencing the symptoms at least once during their lifetime.^10,11^ Other presentations are anaemia, melena, haematochezia, haematemesis and obstruction.

Imaging in Peutz–Jeghers syndrome is performed for diagnosis, identification of complications and surveillance to reduce the risks associated with Peutz–Jeghers syndrome.^1,12^ Regular small bowel surveillance is mainly performed for identifying and reducing the risk of complications, especially intussusception and intestinal malignancy associated with Peutz–Jeghers syndrome. Recently developed techniques such as capsule endoscopy, CT and MR enteroclysis and enterography have an important role in the management of Peutz–Jeghers syndrome.^1,12^

Most studies also indicate that hamartomatous polyps in Peutz–Jeghers syndrome can cause multiple intussusceptions, and may also be associated with malignancies. To the best of our knowledge, this is the first reported case of synchronous colocolic intussusceptions in a patient with Peutz–Jeghers syndrome. Imaging plays a key role in the diagnosis of acute abdominal emergencies and surveillance in patients with Peutz–Jeghers syndrome.

## Learning points

1. Adult intussusceptions, although rare, are a common abdominal emergency in patients with polyposis syndrome and, although rare, may also occur synchronously, especially in the small bowel.
2. CT scan plays a crucial role in diagnosing these cases.
3. Synchronous colocolic intussusceptions, as seen in our case, however, has not been reported before in association with polyposis syndromes.

## Consent

Written informed consent was obtained for publishing this case report.
